# Society Notes

**Published:** 1904-12

**Authors:** 


EAST TEXAS MEDICO-CHIRURGICAL ASSOCIATION held its ninth semi-annual meeting, in joint session with the Houston County Medical Society, at Crockett, Texas, December 1st and 2d (inst.), Dr. E. D. Link, Palestine, President. A splendid program was carried out, followed by a banquet. Some of the papers will appear in the "Red Back" later.

